# Kinetic and isotherm studies of adsorption and biosorption processes in the removal of phenolic compounds from aqueous solutions: comparative study

**DOI:** 10.1186/2052-336X-11-29

**Published:** 2013-12-19

**Authors:** Abdolmajid Gholizadeh, Majid Kermani, Mitra Gholami, Mehdi Farzadkia

**Affiliations:** 1 Department of Environmental Health Engineering, School of Public Health, Tehran University of Medical Sciences, Tehran, Iran; 2 Department of Environmental Health Engineering, School of Public Health, Iran University of Medical Sciences, Tehran, Iran; 3 Center for Air Pollution Research (CAPR), Institute for Environmental Research (IER), Tehran University of Medical Sciences, Tehran, Iran; 4 Center for Water Quality Research (CWQR), Institute for Environmental Research (IER), Tehran University of Medical Sciences, Tehran, Iran; 5 Center for Solid Waste Research (CSWR), Tehran University of Medical Sciences, Tehran, Iran

**Keywords:** Phenolic compounds, Biosorption, Adsorption, Aqueous solutions

## Abstract

The phenolic compounds are known by their carcinogenicity and high toxicity as well as creating unpleasant taste and odor in water resources. The present study develops a cost-effective technology for the treatment of water contaminated with phenolic compounds, including Phenol (Ph), 2-chlorophenol (2-CP), and 4-chlorophenol (4-CP). So, two sorbents, rice bran ash (RBA) and biomass of brown algae, *Cystoseiraindica*, were used and results were compared with the commercially granular activated carbon (GAC). The phenolic compounds were determined using a high performance liquid chromatography (HPLC) under batch equilibrium conditions. The effects of contact time, pH, initial adsorbate concentration, and adsorbent dosages on the removal efficiency were studied. The adsorption data were simulated by isotherm and kinetic models. Results indicated that RBA and GAC had the lowest efficiency for the removal of 2-CP, while the order of removal efficiency for *C. indica* biomass was as follows: 2-CP > 4-CP > phenol. The efficiency of GAC was higher than those of other adsorbents for all of the phenolic compounds. Furthermore, the adsorption capacity of RBA was found to be higher than that of *C. indica* biomass. The optimal initial pH for the removal of phenol, 2-CP and 4-CP was determined to be 5, 7, and 7 for RBA, GAC, and algal biomass, respectively. Kinetic studies suggested that the pseudo-second order best fitted the kinetic data.

## Introduction

In the recent decades, phenolic pollution has arisen as a serious problem in terms of its carcinogenicity and high toxicity as well as creating unpleasant taste and odor in water resources. The main sources of phenol and chlorophenols are refineries; petrochemical industries [1-3]; industrial resins; plastics; adhesives; rubber, iron, steel, aluminum, pulp, and paper industries; pesticides; fungicides; bactericides; herbicides; fungicides; medical and health products, including oils, softeners, mouthwash, and eye and ear drops; tannins; electrical industries; and, finally, paint industries [4-7]. United State Environmental Protection Agency (USEPA) has listed phenolic compounds as precedence compounds [6]. Because of low biological degradability, high toxicity, and high ecological endurance of phenolic compounds, wastewaters containing these compounds can pose many problems [8]. The guideline proposed by WHO for phenol concentration in drinking water is <2 μg/L [9,10]. Some physicochemical processes have been developed for the removal of phenolic compounds, including adsorption, extraction by chemical solvents, air stripping, freezing and crystallization, chemical oxidation, wet oxidation, and advanced oxidation processes (AOPs) [11-13]. Application of such techniques poses high operational costs due to the necessity of continuous addition of chemical compounds, and causes further environmental damages [14]. noteworthy a wide range of sorbents have been examined for the removal of phenol from aqueous solutions, among which activated carbon is accepted as the most powerful adsorbent, thus being extensively used [15]. More recently, different types of natural, low-cost sorbents have been investigated. Agricultural wastes and seaweeds have been considered as one of the most promising groups of adsorbents [16].

The rice bran is an agricultural waste and ninety six percent of the rice is produced in developing countries [17], particularly in South-western Asian countries such as Iran [18]. The main components of rice bran are carbon and silica, both of which posse high porosity and large surface area. It is noteworthy that silica is concentrated in bran by burning [19].

Biosorption is also a substitute technology which describes as the removal of external compounds from aqueous solutions by passive binding to non-living biomass, including fungi, bacteria, yeast, micro and macro algae [20-22]. General mechanisms involved in biosorption are complex surface adsorption, ion exchange, chelation of fine sediments in the micropore, and oxidation reactions [23-25]. Generally, the cellular walls of Phaeophyta (brown seaweed) constitute a fibrillar skeleton and an amorphous matrix. The outer layer is an amorphous matrix that is linked to the fibrillar skeleton (mainly cellulose) via hydrogen bonding [26]. The alginate contributes to the litheness and resistance of the cellular wall. The biosorption uptake is directly related to the presence of carboxyl groups in the alginate polymer. its biomass also acts as ion exchange resins [27,28]; thus it can attract complex compounds such as phenolic compounds, which are negatively charged. Some brown algae, such as *Padinapavonica* and *Sargassummuticum, Pelvetiacanaliculata* and etc. have been considered for biosorption process [13,29,30].

Therefore the present study aimed to evaluate the efficiencies of brown algae, i.e. *Cystoseiraindica*, and rice bran for the removal of phenol, 2-chlorophenols, and 4-chlorophenol from aqueous solutions, and to compare their efficiencies with that of the commercially available GAC. For this purpose, kinetic and isotherm studies have been done.

## Material and methods

### Preparation of biomass and media

#### Preparation of the biosorbent (C. indica biomass)

*C. indica* brown alga was obtained from the Persian Gulf on the coast of Chabahar, Iran. Then, it was rinsed several times with deionized water, and then dried in the ambient air and kept in an oven at 60°C overnight. The dried biomass was grounded in the laboratory blender and sorted by using the standard sieves. In order to improve the stability and the biosorption capacity of the algal biomass, it was chemically pretreated with CaCl_2_. This can lead to the accessibility of more anionic sites, due possibly to the removal of surface impurities [13,31]. For the pretreatment process, the batch of biomass with a particle size of 0.5-2 mm was selected. Pretreatment of the biomass was carried out as follows: the sample of the biomass was treated with 0.1 M CaCl_2_ at pH = 5 (40 g biomass per liter of solution) for 3 h at 25°C under slow stirring (150 rpm). Then, the pretreated biomass was rinsed, dried in an oven at 60°C overnight, and kept in a desiccator for future usage.

#### Rice bran ash (RBA)

The rice bran was taken from Babol, north of Iran. In order to prepare the rice bran ash (RBA), rice bran was crushed, sieved, and thoroughly rinsed with distilled water, and then dried at 105°C for 2 h until reaching a constant weight. Afterwards, it was burned at 400°C temperature (RBA-400) for 2 h in a muffle furnace. Finally, the rice bran ashes were powdered and stored in desiccator [18,32].

#### Granular activated carbon (GAC)

The commercially available GAC was obtained from Merck Co., Germany, and used without further purification.

#### Phenolic solutions

Phenol, 2-chlorophenol, and 4-chlorophenol were obtained from Merck Co., Germany, and used without further purification. A stock solution was prepared by dissolving 1.0 g of Ph, 2-CPh, and 4-CPh in 1.0 lit of purified water. The concentrations of phenolic compounds were in the range of 50–400 mg/l for the experiments.

### Surface morphology of the biosorbent and adsorbents

Surface morphology of the selected adsorbents/biosorbent was investigated using scanning electron microscopy (SEM) (Stereo Scan LEO, Model-400). The sample was placed on the aluminum tub and coated with gold to improve the electron conductivity and image quality. Then, the samples were vacuumed for 5–10 min prior to the analysis.

### Analysis and biosorption and adsorption studies

Biosorption and adsorption of ph, 2-CP, and 4-CP by algal biomass, RBA, and GAC were investigated in a batch reactor (inner Erlenmeyer’s volume: 100 ml). Concentrations of the phenolic compounds were measured in the aqueous phase by a high performance liquid chromatography (HPLC) instrument (CECIL Co., CE4200, England). The chromatographic measurement of phenolic compounds was carried out using a HICHROM-HI-5C18-4371, HPLC column (150 mm × 4.6 mm inner diameter (i.d.); 5 μm) protected with a guard column (HICHROM; 20 mm × 4.0 mm i.d.). All sample solutions used in the chromatographic studies were pre-filtered by a 0.45 μm PTFE Hydrophilic Gamma sterile filter (25 mm). HPLC mobile phases A and B were Acetonitrile and 0.05 M Phosphate buffer (KH_2_PO_4_), respectively. The mobile phases were also filtered prior to use. The chromatographic determination was performed using a gradient at 1.5 ml/min flow-rate (in 0–20 min); phase A from 55% to 100% and phase B from 45% to 0%, return to phase A and B 35% and 65% in 15 min, respectively). The sample injection volume was 20 μL. The UV-visible detector was set at 270, 274 and, 280 nm for the measurement of phenol, 2-CP, and 4-CP, respectively.

Preliminary kinetic tests were carried out in order to determine the equilibrium time for each adsorbate. For this purpose, 0.4 g of *C. indica* biomass, RBA, and GAC were separately added to 40 mL of each phenolic solution having a concentration of 50 mg/L and an initial pH of 5, and then poured into 100-mL Erlenmeyer flasks. The flasks were placed on a rotating shaker (HeidolphUnimax 2010, Germany) at 150 rpm constant agitation and 21 ± 2°C. The pH of the solutions was adjusted at 5 ± 0.2 during the contact period (5–300 min) using 0.01 N HCl and 0.01 N NaOH. Then, the samples were filtered through filter papers (Whatman, No. 41 Ashless), and the concentrations of the phenolic compounds were analysed. Finally, as mentioned in Section 3.8, the kinetic equations were obtained. In addition, the effect of pH (2–11), initial phenol, 2-CP, and 4-CP concentrations (50–400 mg/L), and sorbent dosage (0.1-0.4 g/40 mL) on the removal efficiency were studied.

Equilibrium Experiments were conducted to determine the equilibrium isotherms for C.indica biomass, RBA, and GAC in the optimal operating conditions in terms of contact time, pH, and concentrations of the phenolic compounds. Finally, the removal efficiency and the adsorption capacity were determined.

In order to describe the relationship between the amount of each phenolic compound and the adsorbents/biosorbent dosages, Langmuir, Freundlich, and Temkin models were applied. Kinetic data were also fitted to the pseudo-first order, pseudo-second order, and intra-particle diffusion models.

### Validity of the kinetic and isotherm models

The applicability of the kinetic and isotherm models was confirmed by calculating the normalized standard deviation (NSD) and average relative error (ARE) factors between the experimental data and the model estimates. These were then used to predict the sorption behaviors of phenol, 2-CP, and 4-CP. The mathematical equations of NSD and ARE are given below:

(1) $$\mathrm{NSD}\left(\%\right)=100\sqrt{\frac{1}{N-1}\sum_{i}^{N}{\left[\frac{q_{i,\text{exp}}-q_{i,\text{cal}}}{q_{i.\text{exp}}}\right]}^{2}}$$

(2) $$\mathrm{ARE}=\frac{100}{N}\sum_{i=1}^{n}{\left|\frac{q_{i,\text{exp}}-q_{i,\text{cal}}}{q_{i.\text{exp}}}\right|}_{i}$$

where, q_i,exp_ and q_i,cal_ (mg/g) are experimental and calculated mass of the phenolic compounds adsorbed/bisorbed by RBA, GAC, and *C. indica* biomass at time t, and N is the number of measurements made. Smaller NSD and ARE values imply more accurate estimations [33,34].

## Results

### Scanning electron microscope (SEM) studies

**Figure 1:**
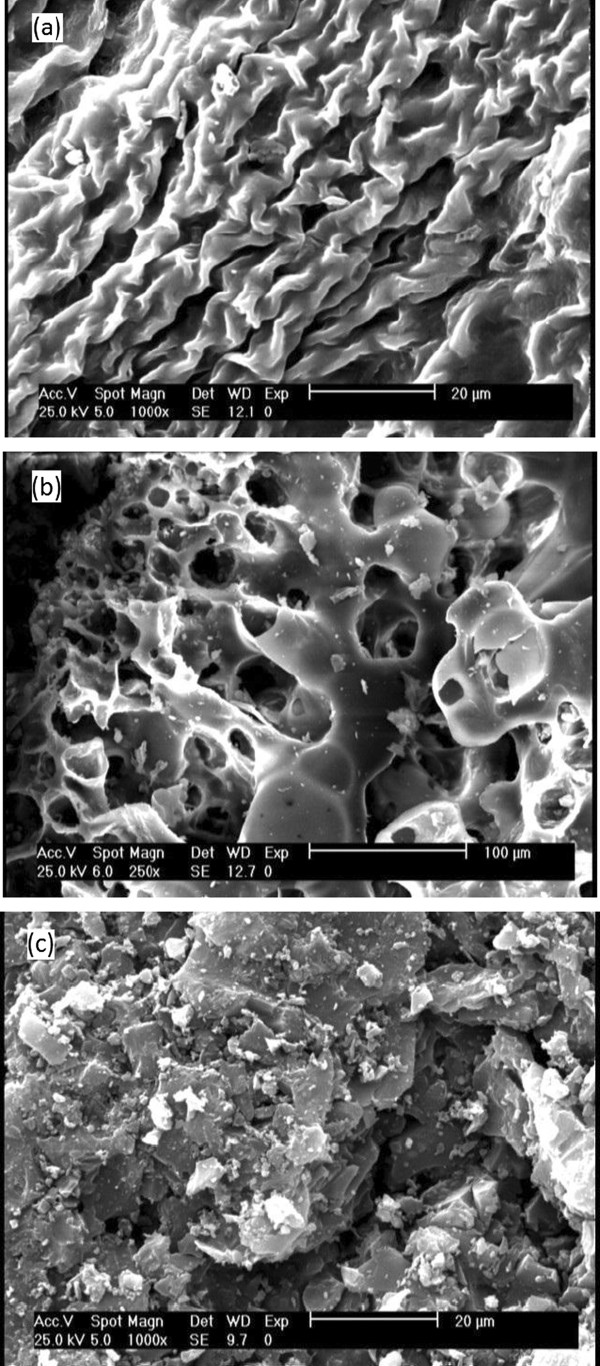
**SEM images of (a) *C. indica *** **biomass (1000×), (b) RBA (250×) and (c) GAC (1000×).**

Micrographs of *C. indica* biomass, RBA, and GAC surfaces recorded by a software-controlled digital SEM are shown in Figure 1(a-c). The SEM of *C. indica* biomass (Figure 1(a)) showed no particular crystalline structure and appeared as nearly spherical. Figure 1(b) illustrates the surface texture and porosity of RBA, with holes and small openings on the surface, thereby increasing the contact area, which facilitates pore diffusion during the adsorption/biosorption process.

### Effect of contact time on the biosorption/adsorption process

**Figure 2:**
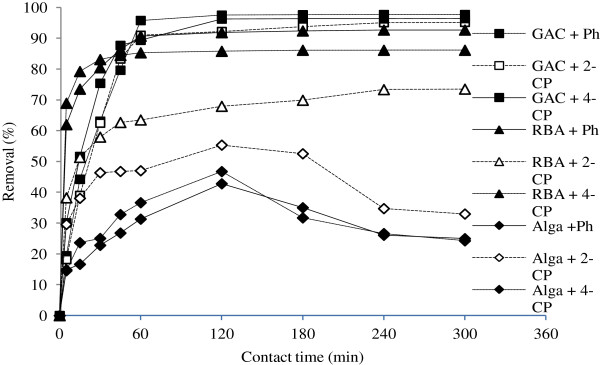
Effect of contact time on the removal of phenol, 2-CP and 4-CP by RBA, GAC and algal biomass.

Adsorption/biosorption of Ph, 2-CP, and 4-CP by *C. indica* biomass, RBA, and GAC were evaluated in an initial adsorbate concentration of 50 mg/L, a pH of 5, and 0.4 g of each adsorbent/biosorbent. Results indicated that the contact time influences the sorption process; the adsorption capacity of all of the phenolic compounds (Ph, 2-CP, and 4-CP) increases with time and reaches a constant value at a certain time, after which no more phenols are removed from the solution. The time required by the phenolic compounds to reach equilibrium condition was found to be 240 and 60 min for RBA and GAC, respectively. Related data is depicted in Figure 2. Sorption of the phenolic compounds was noticeable at the beginning of the process. However, the biosorption process on the algal biomass was different; it reached the equilibrium when the sorption rate was equal to desorption rate, i.e. after 120 min.

RBA and GAC indicated higher adsorption capacities for phenol and 4-chlorophenol than for 2-chlorophenol. However, the order of biosorption capacity for the algal biomass was as follows: 4-CP *>* 2-CP *>* phenol.

### Effect of pH on the biosorption process

**Figure 3:**
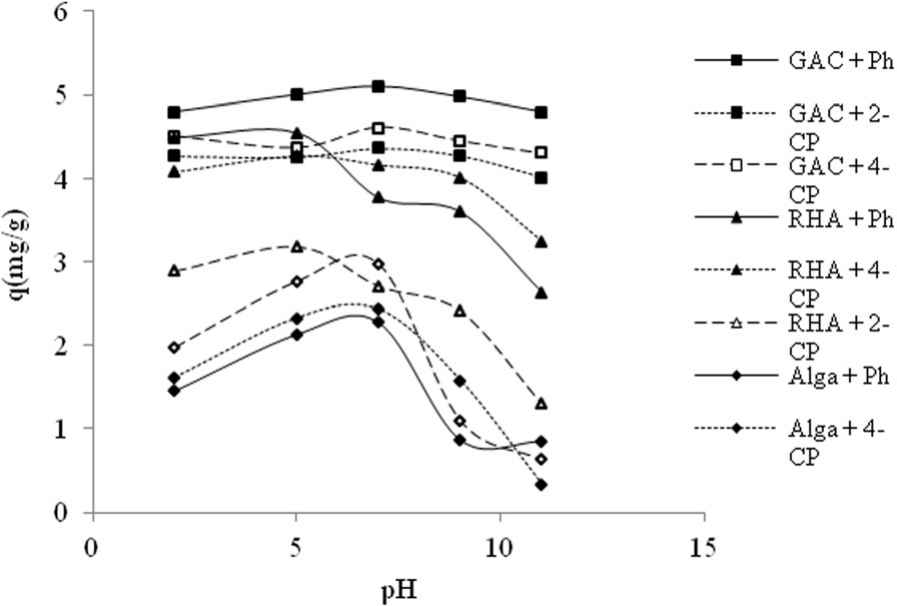
Effect of solution pH on the removal of phenol, 2-CP and 4-CP by RBA, GAC and algal biomass.

In order to optimize the pH to achieve maximum removal efficiency, experiments were conducted in a wide range of pH, from 3 to 11. Experimental results indicated that phenol, *2*-CP, and 4-CP were more effectively removed at pH = 7, 5, and 7 by algal biomass, RBA, and GAC, respectively; however, the removal efficiency decreased on either side of these pH values (Figure 3), Table 1.

**Table 1:** Comparison of various medium for the adsorption of phenol and its derivatives

**Adsorbent**	**Solute**	**pH**	**Equilibrium time (min)**	**Capacity (mg/g)**	**Reference**
*N-methylacetamide-modified hypercrosslinked resin*	Phenol	6.3	200	170	[35]
*AC (from kraft black liquor)*	Phenol	6	-	227	[36]
*AC (from kraft black liquor)*	2,4,6-TCP	6	-	476	
*chitosan–calcium alginate blended beads*	phenol	7	240	51	[12]
*chitosan–calcium alginate blended beads*	*o*-chlorophenol	7	240	41.5	
*Schizophyllum commune fungus*	Phenol	5	120	42	[1]
*Schizophyllum commune fungus*	2-CP	5	120	46	
*Schizophyllum commune fungus*	4-CP	5	120	48	
*chitosan-conjugated thermo-responsive polymers*	phenol	-	120	-	
*chitosan-conjugated thermo-responsive polymers*	4-methylphenol	-	120	-	[15]
*chitosan-conjugated thermo-responsive polymers*	4-methoxyphenol	-	120	-	
*chitosan-conjugated thermo-responsive polymers*	4-CP	-	120	-	
*Sargassummuticum*	Phenol	-	8–10 h	2*.*8	[13]
*Sargassummuticum*	2-CP	6	8–10 h	22*.*0	
*Sargassummuticum*	4-CP	6	8–10 h	24*.*3	
*Posidoniaoceanica (L.) seagrass*	2,4-DCP	5	1440	-	[37]
*Clay (quartz, kalinite, etc.) 50 mm*	Phenol	6.5	-	30.3	
*SDS-alumina*	Phenol	6.7	-	6.6	[38]
*Paper mill sludge*	2-CP	7	180	0.34	[39]
*Paper mill sludge*	3-CP	8	180	1.04	
*Paper mill sludge*	4-CP	8	180	0.99	
*Paper mill sludge*	2-NP	8	180	0.12	
*Dried activated sludge*	2-CP	1	-	281.1	[40]
*Dried activated sludge*	4-CP	1	-	287	
*Bentonite*	Phenol	5	-	1.7	[41]
*RBA*	Phenol	5	240	4.63	*
*RBA*	2-CP	5	240	3.66	*
*RBA*	4-CP	5	240	4.30	*
*C. indica biomass*	Phenol	7	120	2.14	*
*C. indica biomass*	2-CP	7	120	2.77	*
*C. indica biomass*	4-CP	7	120	2.34	*
*GAC*	Phenol	7	60	4.85	*
*GAC*	2-CP	7	60	4.28	*
*GAC*	4-CP	7	60	4.47	*

* Current study.

### Effect of initial concentration of the phenolic compounds

**Figure 4:**
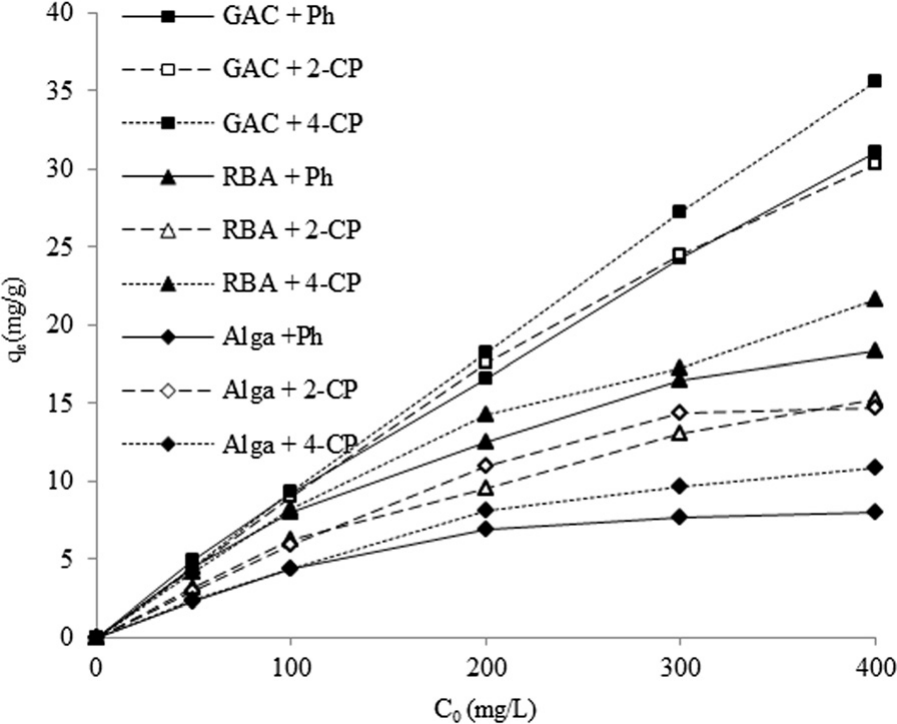
**Effect of initial phenolic concentration on adsorption/biosorption capacity by RBA, *C. indica *** **and GAC.**

As shown in Figure 4, increasing of the initial concentration of the phenolic compounds led to an increase in the adsorbed mass of the pollutants on all of the sorbents. It is clear that the removal of phenols by *C. indica* biomass, RBA, and GAC depends on the concentration of the phenolic compounds. Maximum adsorption/biosorption capacities for Ph, 2-CP, and 4-CP were attained at 400 mg/L for the selected sorbents.

### Effect of adsorbent/biosorbent dosage on the removal of the phenolic compounds

**Figure 5:**
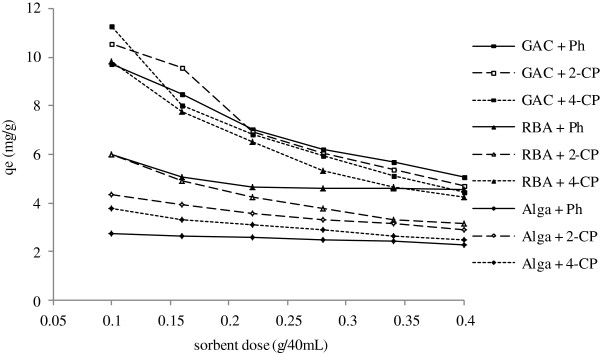
**Effect of different adsorbent/biosorbent dose on adsorption/biosorption capacity by RBA,** ***C. indica *** **and GAC.**

The effect of sorbent dosage on the sorption of the selected phenolic compounds is illustrated in Figure 5. The removal of phenolic compounds is dependent on the mass of RBA, GAC, or algal biomass present in the solution, which increases with increasing of adsorbent dosage, especially more rapidly in the initial stages, and then remains almost constant.

### Isotherm studies

The adsorption isotherm is an equation that shows the transmission of adsorbate from solution phase to the adsorbent phase at equilibrium condition. Langmuir, Freundlich, and Temkin isotherms were used to evaluate the experimental results.

The linearized Freundlich adsorption isotherm is in the following form [42]:

(3) $$\text{log}\left(q_{e}\right)=\text{log}\left(K_{f}\right)+\frac{1}{n}\text{log}\left(C_{e}\right)$$

where, K_F_ and n constants indicate adsorption capacity and adsorption intensity, respectively. Lower fractional values of n [0 < n < 1] indicate that weak adsorptive forces are effective on the surface of the sorbents;

Langmuir adsorption model describes the monolayer adsorption of the adsorbate on a homogeneous adsorbent surface which the linear form of it [type (I)] can be described as [43]:

(4) $$\frac{C_{\mathrm{eq}}}{q_{\mathrm{eq}}}=\frac{1}{q_{m}b}+\frac{C_{\mathrm{eq}}}{q_{m}}$$

where, q_m_ is the maximum amount of adsorption (mg/g), and b is the adsorption equilibrium constant (L/mg).

Regarding to the Temkin model the heat of adsorption of all of the molecules in the layer would decrease linearly with coverage. The linear form of the Temkin isotherm is represented by the following equation [33]:

(5) $$q_{e}=q_{m}{\text{lnK}}_{T}+q_{m}{\text{lnC}}_{e}$$

where, K_T_ is the binding constant representing the maximum binding energy (L/mg).

The applicability of the isotherm equation to describe the adsorption process was assessed by the correlation coefficients, R^2^, NSD, and ARE values.

According to the R^2^, NSD, and ARE values (Table 2), it can be concluded that the Freundlich isotherm model best fitted the experimental data best for the adsorption/biosorption of 4-CP by all of the sorbents. Values of Langmuir constants, q_max_ and b, obtained from the linear plot of C_e_/q_e_ against C_e_, their correlation coefficient (R^2^), NSD, and ARE were also obtained (Table 2). Data revealed that the Langmuir isotherm best fitted the experimental data for the sorption of Ph by all of the applied sorbents. However, 2-CP sorption by RBA and GAC and *C. indica* best fitted the Freundlich, Langmuir, and Freundlich isotherms, respectively.

**Table 2:** **Isotherms parameters by linear regression method for the sorption of Ph, 2-CP and 4-CP by RBA, GAC and *C. indica *** **biomass**

**Models**	**Sorbent type**	**Ph**					**2-CP**					**4-CP**				
		***q***_**m**_	***b***	**R**^**2**^	**NSD**	**ARE**	***q***_**m**_	***b***	**R**^**2**^	**NSD**	**ARE**	***q***_**m**_	***b***	**R**^**2**^	**NSD**	**ARE**
Langmuir	RBA	5.90	0.28	0.94	13.57	2.332	51.81	0.002	0.147	176.36	160.97	27.70	0.02	0.69	9.59	4.20
	GAC	10.30	0.29	0.96	1.04	1.31	12.77	0.166	0.90	3.3	3.07	20.48	0.042	0.75	11.23	8.6
	*C. indica*	3.95	0.05	0.98	1.49	1.17	9.78	0.019	0.92	4.61	2.17	18.31	0.006	0.05	2.32	1.95
models	sorbent type	k_F_	n	R^2^	NSD	ARE	k_F_	n	R^2^	NSD	ARE	k_F_	n	R^2^	NSD	ARE
Freundlich	RBA	3.71	10.3	0.44	7.81	0.09	0.13	1.9	0.96	4.54	0.025	0.94	1.4	0.95	6.86	0.050
	GAC	1.93	5.04	0.88	2.62	2.01	3.28	2.74	0.86	4.25	3.98	1.40	1.57	0.94	1.6	1.51
	*C. indica*	0.69	2.75	0.94	1.59	439.5	0.43	1.61	0.98	2.07	1.69	0.16	1.20	0.98	1.88	1.4
models	sorbent type	K_T_	*q*_m_	R^2^	NSD	ARE	K_T_	*q*_m_	R^2^	NSD	ARE	K_T_	*q*_m_	R^2^	NSD	ARE
Temkin	RBA	1200	0.49	0.42	8.08	0.49	0.09	4.61	0.93	7.33	1.23	0.28	4.62	0.92	11.4	0.32
	GAC	0.02	0.6	0.82	9.02	8.5	0.515	0.31	0.81	6.4	5.55	274	0.195	0.88	4.11	3.7
	*C. indica*	0.91	0.46	0.93	119.3	118.5	0.17	2.22	0.96	36.4	31.1	0.103	2.53	0.97	264	240

### Kinetic models

The pseudo-first order equation can be written as follows [42]:

(6) $$\ln\left(q_{e}-q\right)=\ln\left(q_{e}\right)-\frac{K_{1}t}{2.303}$$

where, q_e_ is the amount of selected compounds adsorbed at equilibrium (mg/g), q_t_ is the amount of selected compounds adsorbed at time t (mg/g), k_1_ (1/min) is the pseudo-first order rate constant.

The pseudo-second order model is as the following equation [33]:

(7) $$\frac{t}{q}=\frac{1}{K_{2}q_{e}^{2}}+\frac{1}{q_{e}}t$$

where, k_2_ (g/mg min) is the rate constant of the second-order equation.

Also the intra-particle diffusion equation is expressed as follows [34]:

(8) $$q_{t}=K_{\text{int}}t^{1/2}+C$$

where, k_int_ (mg/g min^1/2^) is the rate constant of intra-particle diffusion model and C is a constant that indicates the thickness of the margin layer; i.e. the larger the intercept, the greater the boundary layer effect.

Linear expressions of these kinetic equations are presented in Table 3. These strongly suggesting that the sorption of Ph, 2-CP, and 4-CP by RBA, *C. indica*, and GAC best fits a pseudo-second order kinetic model.

**Table 3:** **Kinetic models parameters by linear regression method for the sorption of Ph, 2-CP and 4-CP by RBA,** ***C. indica *** **and GAC at 50 mg/L initial Phenols concentration**

**Models**	**Sorbent type**	**Ph**					**2-CP**					**4-CP**				
		***q***_**e**_	***K***_***1***_	**R**^**2**^	**NSD**	**ARE**	***q***_**e**_	***K***_***1***_	**R**^**2**^	**NSD**	**ARE**	***q***_**e**_	***K***_***1***_	**R**^**2**^	**NSD**	**ARE**
Pseudo first order																
	RBA	1.55	0.02	0.94	79.7	74.6	1.97	0.01	0.88	66.5	60.6	0.58	0.026	0.83	105.9	99.9
	GAC	4.28	0.04	0.94	23.2	15.7	7.25	0.02	0.93	55.3	46.4	3.3	0.03	0.89	73.5	70.9
	*C. indica*	1.24	0.003	0.37	82.3	74.7	0.59	0.0004	0.004	104.6	98.6	1.2	0.002	0.14	91.2	84.7
models	sorbent type	*q*_e_	K_2_	R^2^	NSD	ARE	*q*_e_	K_2_	R^2^	NSD	ARE	*q*_e_	K_2_	R^2^	NSD	ARE
Pseudo second order	RBA	4.73	0.06	0.99	4.32	0.38	3.73	0.03	0.99	10.62	3.22	4.38	0. 20	1	1.09	0.4
	GAC	5.2	0.012	0.99	13.2	7.02	4.76	0.012	0.99	4.04	3.54	5.03	0.021	0.99	9.2	8.8
	*C. indica*	1.32	0.12	0.95	20.9	15.5	1.70	0.04	0.95	46.94	38.9	1.28	0.06	0.96	36.5	30.5
models	sorbent type	C	*K*_*int*_	R^2^	NSD	ARE	C	*K*_*int*_	R^2^	NSD	ARE	C	*K*_*int*_	R^2^	NSD	ARE
Intra particle diffusion	RBA	2.34	0.18	0.52	15.4	7.02	1.46	0.158	0.67	14.3	6.35	2.58	0.144	0.37	16.1	7.27
	GAC	2.5	3.06	0.68	15.1	10.5	1.06	0.24	0.74	66.6	60.2	1.56	0.24	0.70	32.1	21.3
	*C. indica*	0.95	0.04	0.26	28.02	23.2	2.06	0.004	0.002	70.2	19.8	1.22	0.02	0.08	95.3	45.2

## Discussion

Morphologic studies were developed by SEM set. The micrograph of GAC indicated the rough, heterogeneous, and porous nature of the adsorbent surface, making the adsorption of phenol molecules on different parts of adsorbent material possible. The inner surface appeared to have the same type of texture and morphology as the outer surface.

RBA and GAC indicated higher adsorption capacities for phenol and 4-chlorophenol than for 2-chlorophenol. However, the order of biosorption capacity for the algal biomass was as follows: 4-CP *>* 2-CP *>* phenol.

The aqueous solubility of phenol (8.0 g/100 g of water) is higher than those of 2-chlorophenol (2.85 g/100 g of water) and 4-chlorophenol (2.0 g/100 g of water). Compared to phenol, chlorophenols, particularly 4-chlorophenol, showed more affinity for non-aqueous medium, as suggested by their octanol–water partition coefficients (log k_ow_ were 1.46, 2.15, and 2.39 for phenol, 2-chlorophenol, and 4-chlorophenol, respectively). Based on the aqueous solubility and octanol–water partition coefficient, *4*-chlorophenol is expected to be adsorbed at a greater extent than phenol and 2-chlorophenol [12,44], which was verified in the case of *C. indica* biomass. Other studies have also reported that 4-chlorophenol is preferentially adsorbed in a multi-ion system [5,8,13,45]. Nevertheless, the experimental results of RBA and GAC indicated that the order of adsorption capacity was as follows: Ph > 2-CP > 4-CP. The adsorption of phenol, 2-CP, and 4-CP by GAC and RBA at different contact times cannot be correlated with physical parameters such as solubility and octanol–water partition coefficients. To justify the observed behavior, some additional retention mechanisms should be contemplated, including donor-acceptor interactions between the aromatic ring activated by OH^–^, Cl^–^ substituents, the groups on the adsorbents surface, and the interactions of phenols with the GAC and RBA by complex formation [8]. Nadavala et al. [12] and Calace et al. [39] observed similar trends in the adsorption of phenols on chitosan–calcium alginate blended beads and paper mill sludge. More comparative results of some of the previous studies are given in Table 1. Results also indicated that the order of removal of all of the phenolic compounds was as follows: GAC > RBA > *C. indica* biomass. GAC exhibited the maximum adsorption capacity; 4.85 mg/g of phenol, 4.28 mg/g of *2*-chlorophenol, and 4.47 mg/g of 4-chlorophenol. The same trend has been previously reported for the sorption of 2,4-dichlorophenol (2,4-DCP) by *Posidoniaoceanica* (L.) seagrass [37].

The effect of pH of the solution on the removal efficiency could be explained; Ph, 2–CP, and 4-CP all act as weak acids and their degradation in the solution is highly dependent on pH [44,46,47]. Experimental results of pH effect indicated that phenol, *2*-CP, and 4-CP were more effectively removed at pH = 7, 5, and 7 by algal biomass, RBA, and GAC, respectively. The effect of pH on the removal efficiency could be explained by considering the presence of ionic and molecular forms of phenolic compounds in aqueous solutions. The phenolic compounds considered in this study, i.e. Ph, 2-CP, and 4-CP, have pK_a_ values of 9.9, 8.3, and 9.2 respectively, suggesting that phenol is a weak acid; hence, they only exist as anions at high pH values [1]. Phenol and Chlorophenols are proton donors, so they become anions at a certain pH (%50 in the anion form at pH = pK_a_, which varies from 4.7 to 9.4 and decreases with increasing of Cl¯ substitution). In acidic solutions, the molecular form dominates, while in alkaline mediums (pH >pK_a_), the anionic form (phenolate anions) is the predominant species. Therefore, the surface charge on the cells becomes negative, which probably leads to a lower electrostatic attraction between the phenolate anions and the anionic functional groups of cells surface [1,12,40]. Similar findings have been reported by Won et al. [48] on the adsorption of negatively charged reactive dye by D. glutamicum. Some of the previous studies in the literature that evaluated the sorption of almost similar compounds by natural sorbents also indicated the necessity of acidic conditions for achieving optimal conditions for the removal of phenolic compounds [1,49], while the others (Sanjay [3] and Siva Kumar [12]) suggested natural pH for the adsorption of phenols on fly ash and chitosan–calcium alginate blended beads. Wang and Jiang [50] also reported that the high adsorption capacity and removal efficiency of 2-sec-butyl-4,6-dinitrophenol (BDNP) from solutions onto fly ash can be achieved at a pH of 4.0, an adsorbent dosage of 5 g/L, a mesh size of 160–200, a temperature of 20°C, and a contact time of 120 min. Results from other relevant studies in the literature have been presented in Table 1.

Increasing of the initial concentration of the phenolic compounds led to an increase in the adsorbed mass of the pollutants on all of the sorbents. This phenomenon may be related to the driving forces that need to be overcome for the resistances of mass transfer between the aqueous and solid phases. However, at higher concentrations, the available sites of the sorbents become fewer than the moles of phenolic compounds present; hence, their removal efficiency is dependent on the initial concentration. Hameed B. H. [51] reported similar results for the adsorption of phenols on garlic peel. Their results indicated that increasing of the initial concentration of phenolic compounds enhanced the adsorption capacity. Maximum adsorption/biosorption capacities for Ph, 2-CP, and 4-CP were attained at 400 mg/L for the selected sorbents. At low concentrations of phenolic compounds, molecules are attracted more in the outer adsorption sites, while at higher concentrations of phenol and chlorophenols ions, these molecules are necessarily attracted to the inner places. The absorption capacity can be increased by increasing of the initial concentration, due mainly to a higher probability of collision between phenolic compounds and the sorbents, which increases the adsorption capacity [18,52].

The effect of sorbent dosage on the sorption of the selected phenolic compounds showed the removal rate increases with increasing of adsorbent dosage, especially more rapidly in the initial stages, and then remains almost constant. This growth can be attributed to the additional number of adsorption sites, which are resulted from the increase in the adsorbent dosage. On the other hand, the total adsorbed amount of phenols (q_e_) decreases as the adsorbent dose increases. This can be attributed to the aggregation or overlap of the adsorption sites, due primarily to the overcrowding adsorbent particles, which decreases the total adsorbent surface available to the phenols [53,54]. The effect of the algal dose on the removal efficiency of aromatic compounds from water by calcium-pretreated *Sargassummuticum* for different algal doses at pH 1 and an initial concentration of 200 mg/L at room temperature showed that increasing of the biosorbent dose from 0.05 to 0.5 g enhances the removal efficiency, while the biosorption capacity at equilibrium, q_e_ (mg/g*)*, decreases it. The latter result can be explained as a consequence of a partial aggregation, which occurs at high biomass concentrations, leading to decreased active sites [13].

In isotherm studies, the Freundlich constant, *n,* was higher than the unity, suggesting that the adsorption process was favorable under the studied conditions. According to the R^2^, NSD, and ARE values, it can be concluded that the Freundlich isotherm model best fitted the experimental data best for the adsorption/biosorption of 4-CP by all of the sorbents, implying that the surface of all of the sorbents is heterogeneous and multilayer for the 4-CP adsorption/biosorption. Also data revealed that the Langmuir isotherm best fitted the experimental data for the sorption of Ph by all of the applied sorbents. This can be interpreted as the monolayer adsorption of adsorbates onto a homogeneous surface. Moreover, there is a negligible interaction between the adsorbed molecules and adsorption sites that have uniform energies [33]. 2-CP sorption on RBA and GAC and *C. indica* best fitted the Freundlich, Langmuir, and Freundlich isotherms, respectively. This may be due possibly to both homogeneous and heterogeneous distribution of active sites on the surface of the each sorbent.

Linear expressions of kinetic equations showed that low correlation coefficients, as well as high NSD and ARE values confirmed that the linearized form of the pseudo-first order and intra-particle diffusion model do not exhibit a good regression (figures are not shown). However, the correlation coefficients of the linearized form of the pseudo-second order equation were always higher than 0.95. Meanwhile, the NSD and ARE values of this model were lower than the pseudo-first order and intra-particle models. Furthermore, the calculated *q*_e_ value was close to the experimental value, strongly suggesting that the sorption of Ph, 2-CP, and 4-CP by RBA, *C. indica*, and GAC best fits by pseudo-second order kinetic model. This model implies that two reactions, either in series or in parallel, are occurring; the first one is fast and rapidly reaches the equilibrium, while the second is a slower reaction that continues for a long period of time [33,55].

## Conclusion

Natural materials are one of the most promising adsorbents due to their availability, low costs, large specific surface areas, and their chemical and mechanical stability. Therefore, the capabilities of modified algal biomass (*Cystoseiraindica*), carbonized rice bran, and GAC were characterized by isotherm and kinetic studies. The kinetic studies indicated that the adsorption process was extremely fast in all employed sorbents. The experimental results indicated that the two natural sorbents, especially RBA, can be successfully used for the removal of Ph, 2-CP, and 4-CP from aqueous solutions in lieu of the commercial GAC and they can be considered as an innovative and effective process, giving good performances.

## Competing interests

The authors declare that they have no competing interests.

## Authors' contributions

All authors participated in carring out the adsorption and biosorption studies, in preparing the manuscript. All authors read and approved the final manuscript.
